# Prospective monitoring of imaging guideline adherence by physicians in a surgical collaborative: comparison of statistical process control methods for detecting outlying performance

**DOI:** 10.1186/s12911-020-1126-z

**Published:** 2020-05-13

**Authors:** Michael Inadomi, Karandeep Singh, Ji Qi, Rodney Dunn, Susan Linsell, Brian Denton, Patrick Hurley, Eduardo Kleer, James Montie, Khurshid R. Ghani, for the Michigan Urological Surgery Improvement Collaborative (MUSIC)

**Affiliations:** 1 Department of Urology, University of Michigan, NCRC Bldg. 16, 1st Floor, Room 114W, 2800 Plymouth Road, Ann Arbor, MI 48109-2900 USA; 2 Department of Learning Health Sciences, University of Michigan, Ann Arbor, MI 48109 USA; 3 Department of Industrial and Operations Engineering, University of Michigan, Ann Arbor, MI 48109 USA; 4 Comprehensive Urology, Novi, MI 48375 USA; 5 IHA Urology, Ypsilanti, MI 48197 USA

**Keywords:** Monitoring, Quality control, Imaging, Prostate cancer

## Abstract

**Background:**

Systematic, automated methods for monitoring physician performance are necessary if outlying behavior is to be detected promptly and acted on. In the Michigan Urological Surgery Improvement Collaborative (MUSIC), we evaluated several statistical process control (SPC) methods to determine the sensitivity and ease of interpretation for assessing adherence to imaging guidelines for patients with newly diagnosed prostate cancer.

**Methods:**

Following dissemination of imaging guidelines within the Michigan Urological Surgery Improvement Collaborative (MUSIC) for men with newly diagnosed prostate cancer, MUSIC set a target of imaging < 10% of patients for which bone scan is not indicated. We compared four SPC methods using Monte Carlo simulation: p-chart, weighted binomial CUSUM, Bernoulli cumulative sum (CUSUM), and exponentially weighted moving average (EWMA). We simulated non-indicated bone scan rates ranging from 5.9% (within target) to 11.4% (above target) for a representative MUSIC practice. Sensitivity was determined using the average run length (ARL), the time taken to signal a change. We then plotted actual non-indicated bone scan rates for a representative MUSIC practice using each SPC method to qualitatively assess graphical interpretation.

**Results:**

EWMA had the lowest ARL and was able to detect changes significantly earlier than the other SPC methodologies (*p* < 0.001). The p-chart had the highest ARL and thus detected changes slowest (*p* < 0.001). EWMA and p-charts were easier to interpret graphically than CUSUM methods due to their ability to display historical imaging rates.

**Conclusions:**

SPC methods can be used to provide informative and timely feedback regarding adherence to healthcare performance target rates in quality improvement collaboratives. We found the EWMA method most suited for detecting changes in imaging utilization.

## Background

Unnecessary imaging in men with low-risk prostate cancer has been recognized by the American Urological Association as a top priority of its Choosing Wisely campaign [1]. In order to determine when computed tomography and bone scans are appropriate in men diagnosed with prostate cancer, guidelines have been validated and implemented by urologists in the State of Michigan through the Michigan Urological Surgery Improvement Collaborative (MUSIC) [2, 3]. Following an imaging guideline intervention by MUSIC in 2014, rates for non-indicated imaging tests were reduced to low levels with a target rate < 10% achieved, with an increase in adherence to bone scan imaging guidelines from 55.9% of MUSIC urologists to 83.8% [4]. Non-indicated bone scan and non-indicated CT scan rates are highly correlated: among patients who received a non-indicated bone scan also underwent non-indicated CT scan whereas only 3.3% of patients who did not receive a non-indicated bone scan underwent a non-indicated CT scan.

To keep rates of non-indicated imaging tests low, practices within MUSIC are given feedback so corrective action can be taken if imaging utilization increases. Monitoring of practices to identify abnormal variation requires manual input from staff performed on an ad-hoc basis. In many industries, statistical process control (SPC) methods are used to identify outlying performance. Control charts are SPC tools used for visualizing performance of a process relative to desired specification limits. Originally developed for quality control in manufacturing processes, they are used in several areas in healthcare [5–7]. There are a wide variety of control charts to choose from, each providing advantages and disadvantages depending on the outcome being measured [8]. If these methods were applied to urological practice, such tools could rapidly identify significant changes in non-indicated imaging rates and alert practices to outlying behavior at an early stage.

In this context, we set out to determine which SPC method is best suited for monitoring physician performance as it pertains to imaging utilization in a statewide collaborative. We compared several SPC methods’ utility for monitoring rates of non-indicated imaging tests by evaluating their performance on factors such as Type 1 error rate, sensitivity to changes in non-indicated imaging rate, and graphical interpretation. An automated feedback system employing SPC methods would offer the solution to help practices maintain high performance in guideline adherence.

## Methods

### Data source

MUSIC is a physician-led quality improvement collaborative founded in 2011 in partnership with Blue Cross Blue Shield of Michigan. It consists of 44 urology practices in Michigan (comprising approximately 85% of urologists in the state) with the goal of improving the quality and value of prostate cancer care [9]. All men seen in a MUSIC practice for a prostate biopsy or a new diagnosis of prostate cancer are prospectively entered into a clinical registry by trained data abstractors. Currently there are > 50,000 patients in the registry, > 13,000 of whom have undergone imaging for prostate cancer. Past reports describe MUSIC’s work toward decreasing inappropriate imaging in these patients [2–4, 10]. Each MUSIC practice obtained an exemption or approval for collaborative participation from a local Institutional Review Board.

### Study cohort

Our study cohort consisted of all men newly diagnosed with prostate cancer by positive biopsy at a MUSIC practice or first seen at a MUSIC practice following positive biopsy from March 2012 to September 2017 who are or are not eligible for a bone scan according to MUSIC guidelines. To be considered non-indicated for a bone scan by MUSIC imaging appropriateness criteria, the patient must have a Gleason score < 8 and prostate specific antigen (PSA) ≤20 ng/mL. Patients lacking data on whether imaging was performed were excluded, as were patients where a lack of imaging was recorded less than 30 days after the patient’s first positive biopsy or his first encounter with a MUSIC practice. Patients who had received a bone scan ordered by a non-MUSIC practice were excluded if no bone scan was subsequently ordered by a MUSIC practice. Of 26,048 men with prostate cancer in the registry, 18,689 patients were non-indicated for bone scan, of which 1366 (7.3%) underwent a non-indicated bone scan. Bone scan was performed on 4044 of 4983 (81.1%) patients for whom bone scan was indicated.

### SPC methods

The MUSIC imaging appropriateness data has various characteristics that must be accommodated by any control chart used. Quarterly monitoring is desired to avoid providing reports that are too frequent, and the control chart must be capable of accommodating a variable sample size since the number of patients seen by a practice varies.

Adherence to imaging indication guidelines is binary in nature: the care of the patient either followed the guidelines or it did not. This is known as attribute data, and four control chart methods well-suited for this were compared: the p-chart, the weighted binomial cumulative sum (CUSUM), the Bernoulli CUSUM, and the exponentially weighted moving average (EWMA). The p-chart is a simple plot of the sample mean over each time period, where a static upper control limit and lower control limit are used to determine when an alarm is triggered [11]. The weighted binomial CUSUM uses a cumulative summation of the difference between the sample mean and the center line to, much like a golf score relative to par, indicate the direction and magnitude of the deviation from the center line [12]. The Bernoulli CUSUM operates by a principle similar to the weighted binomial CUSUM but is on a patient-by-patient basis rather than using aggregated data over a given time period [13]. The EWMA uses an EWMA statistic derived from all previous sample means of the control chart, which serves as an estimate of the underlying imaging rate, and compares this statistic to dynamic upper and lower control limits that depend on the number of patients in a given time period [11]. An additional file discusses these methods in greater detail (see Additional file 1).

### Monte Carlo simulations

We undertook Monte Carlo simulations to assess control chart sensitivity by comparing average run length (ARL), which is a measure of the length of time necessary to signal a change. The ARL is the average number of periods the chart runs before signaling an alarm; high ARL is desirable in the in-control state because it represents a low false-positive alarm rate while low ARL is desirable in the out-of-control state because it represents a fast response time. While different control charts have different parameters that need to be set for operation, all charts can be directly compared via ARLs; a chart with a higher in-control ARL and a lower out-of-control ARL would have higher resolution and therefore superior performance.

Each chart’s ARL is dependent on the suitability of that chart for evaluation of data in the context of a shift in performance from the in-control state to the expected out-of-control state. In our application of SPC charts, the in-control state is the state in which imaging guideline adherence is high and the out-of-control state is marked by deterioration in imaging guideline adherence, as indicated by a higher rate of non-indicated imaging studies. In order to accurately model these situations, we need realistic values of in-control and out-of-control non-indicated imaging rates. We used MUSIC’s overall pre-intervention and post-intervention non-indicated bone scan rates as the out-of-control and in-control rates of the simulated data to calculate the charts’ ARLs.

Following the 2014 MUSIC imaging guideline intervention, the MUSIC overall non-indicated bone scan rate was 5.9%, an improvement from 11.4% preceding the intervention. Accordingly, 5.9% was used as the in-control non-indicated imaging rate and 11.4% was used as the out-of-control rate of the Monte Carlo simulation in order to approximate actual MUSIC overall performance. Sets of simulated data were generated representing non-indicated scans occurring at both the 5.9% in-control rate and the 11.4% out-of-control rate; for example, each simulated patient in the in-control group had a 5.9% chance of receiving a bone scan. These simulated patients were aggregated into groups of 26 patients per quarter, MUSIC’s mean volume of prostate cancer patients in whom bone scan is non-indicated. The different control charts’ parameters were standardized using the in-control simulated data to provide equivalent in-control performance with a 10% false signal rate over 5 years. Out-of-control simulated data was then processed by each control chart until an alarm was triggered; the length of the run was recorded and the method was repeated for 10,000 runs in order to determine ARL. The charts’ ARLs were also evaluated using simulated data at rates of 6, 7, 8, 9, 10, and 11% to show changes in ARL with smaller deviations from the in-control rate.

### Application of control charts to a MUSIC practice

To assess graphical interpretation of data, a representative MUSIC practice was selected to demonstrate the behavior of the different control charts with data from the actual practice. The practice’s nonindicated bone scan rates were plotted using each control chart method. Chart parameters were determined in a similar manner to that of the Monte Carlo simulations.

## Results

Using Monte Carlo simulation, we found that the EWMA method had the lowest out-of-control ARL and was therefore able to detect changes significantly earlier than the other control chart methodologies (ARL = 5.5 quarters; *p* < 0.001; Table 1). The Bernoulli CUSUM’s ARL of 6.7 quarters and the weighted binomial CUSUM’s ARL of 7.2 quarters were not significantly different from each other (*p* = 0.19). The p-chart had the highest ARL and therefore detected changes significantly later than each other chart methodologies (ARL = 23.0 quarters; *p* < 0.001).

**Table 1:** Control chart performance by methodology

	Average Run Length (Monthly Quarters)
P-chart	23.0
Weighted Binomial CUSUM	7.2
Bernoulli CUSUM	6.7
EWMA	5.5

The out-of-control average run length derived from the Monte Carlo simulation of each control chart methodology. Lower out-of-control ARL is indicative of greater chart sensitivity and is a measure of good performance. Pairwise comparisons between each methodology are different with *p* < 0.001 except between the two CUSUM methodologies, which has *p* = 0.19.

**Fig. 1:**
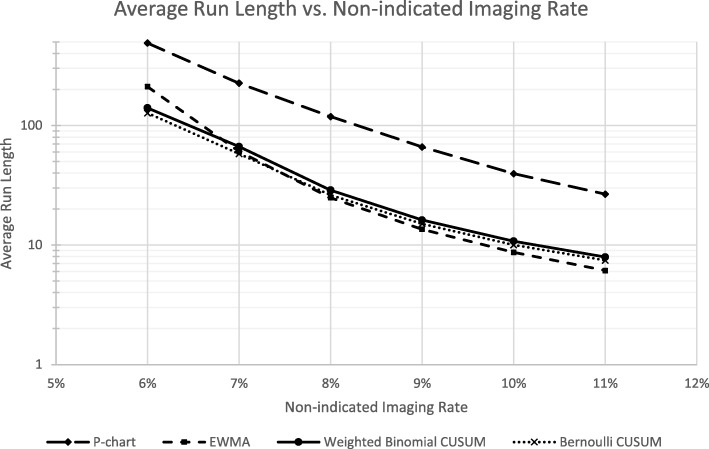
Plot of ARL as it varies with non-indicated imaging rate. EWMA’s steeper slope is indicative of its high resolution. P-chart’s low sensitivity is demonstrated by its high ARL across multiple imaging rates

Comparison of ARL across different out-of-control levels shows that the p-chart had notably worse sensitivity across the different imaging rates as indicated by its high ARLs (Fig. 1). The EWMA method had the lowest ARL at high non-indicated imaging rates while still having a relatively high ARL compared to the CUSUM methods. This suggests that it is sensitive while having a low rate of false signals. Accordingly, it has better resolution, i.e. it has greater positive and negative likelihood ratios, than the other chart methodologies.

**Fig. 2:**
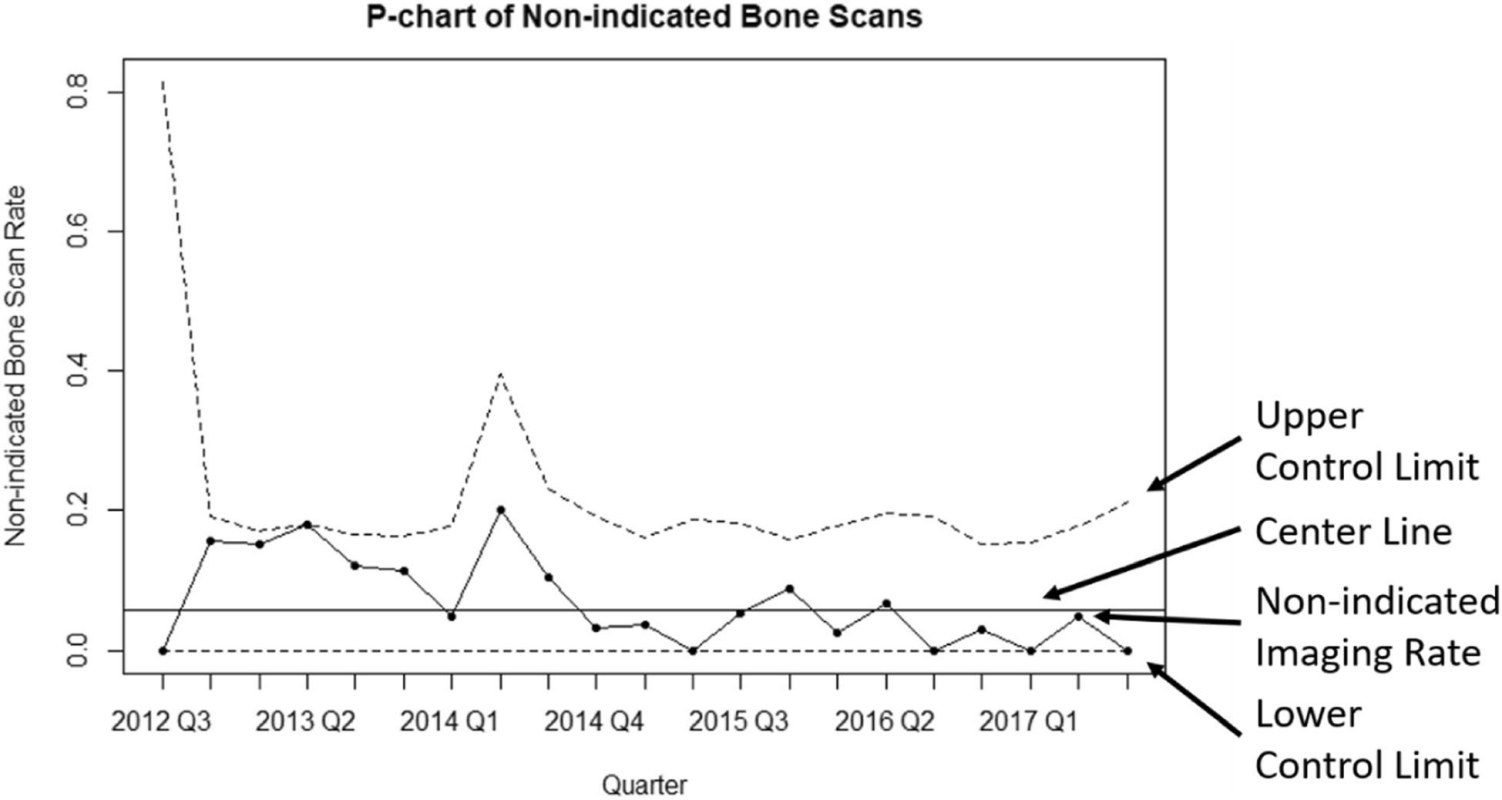
Sample p-chart plot using the data of a single representative MUSIC practice from 2012 to 2017. Note the absence of signal

**Fig. 3:**
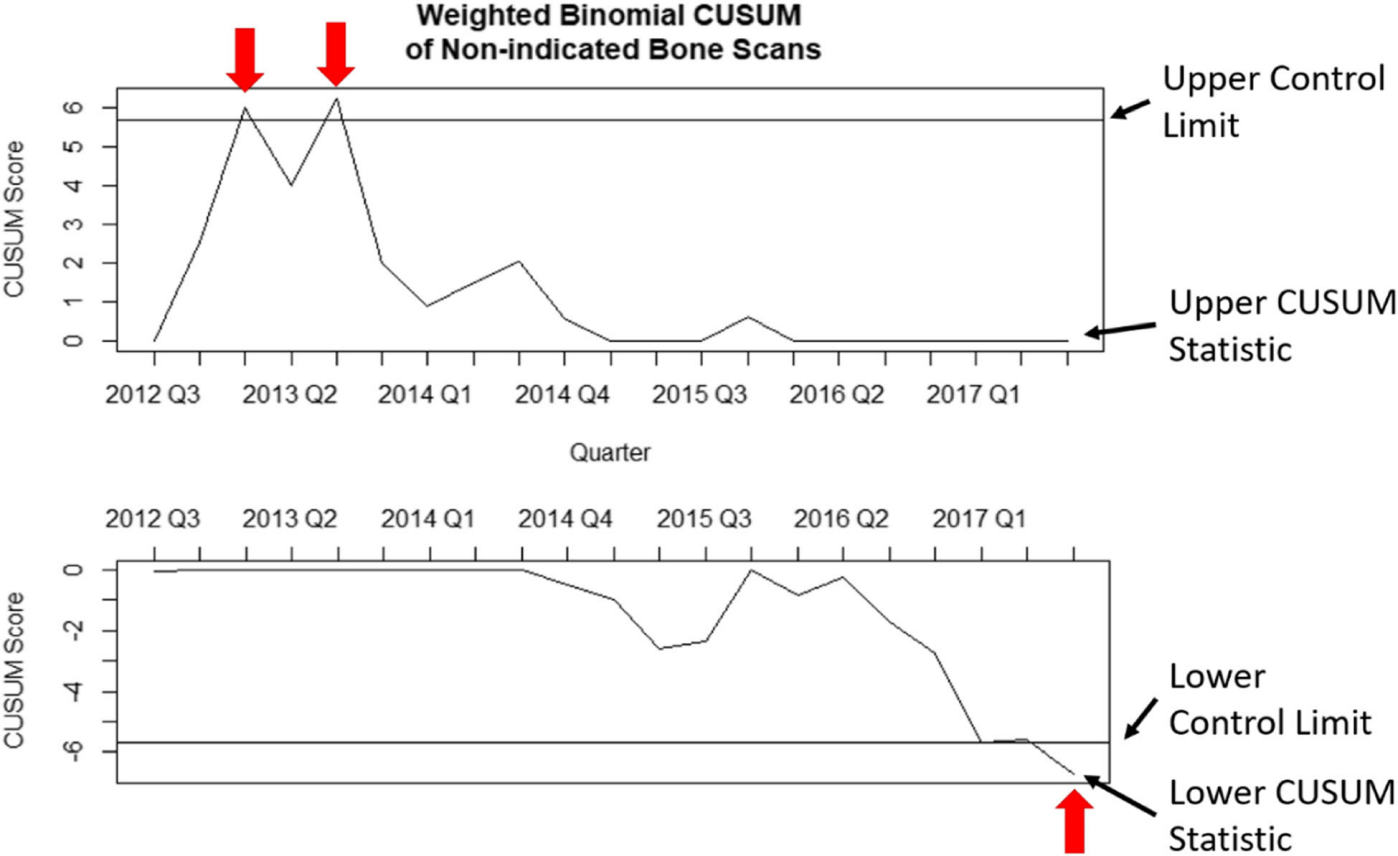
Sample weighted binomial CUSUM plot using the data of the same MUSIC practice as in Fig. 2. Upward deviations in the CUSUM statistic denote increases in the non-indicated bone scan rate while downward deviations denote decreases in the non-indicated bone scan rate. Signal occurs at the arrows, indicating performance is in the out-of-control state

**Fig. 4:**
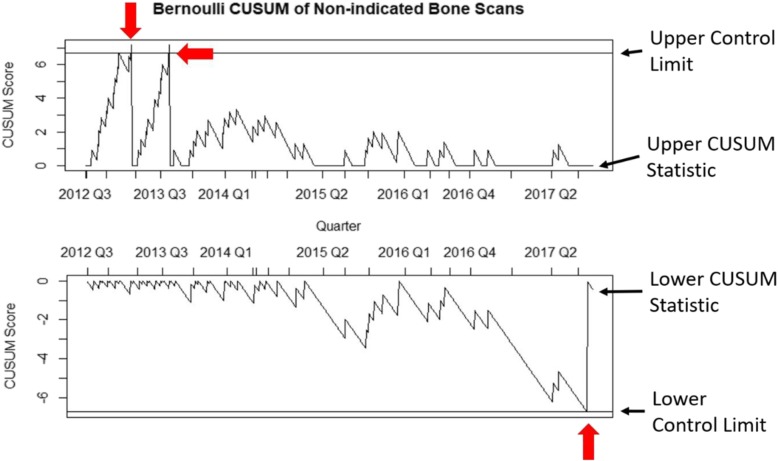
Sample Bernoulli CUSUM plot using the data of the same MUSIC practice as in Fig. 2. Similar to weighted binomial CUSUM, but non-indicated scans are plotted on a patient-by-patient basis

**Fig. 5:**
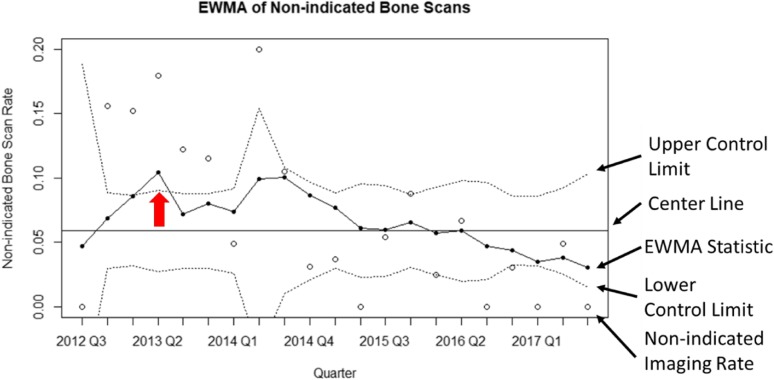
Sample EWMA plot using the data of the same MUSIC practice as in Fig. 2. The EWMA statistic is a weighted average of the non-indicated bone scan rate which gives recent measurements greater weight

The p-chart was the easiest to interpret graphically, followed in order by the EWMA, the weighted binomial CUSUM, and the Bernoulli CUSUM (Figs. 2, 3, 4 and 5). The p-chart’s use of each quarter’s non-indicated historical imaging rate is simpler than the calculated statistics used by the other charts. While the p-chart and EWMA both display data and control limits with a clear relation to the non-indicated imaging rate, the CUSUM charts’ use of a dimensionless CUSUM statistic is more difficult for the untrained viewer to make sense of. The Bernoulli CUSUM has an unconventional appearance, as its patient-by-patient charting results in variation in the spacing of quarterly periods along the horizontal axis.

## Discussion

This study demonstrates EWMA control chart methodology is preferred for monitoring rates of non-indicated imaging tests in a statewide collaborative. We found greater ease of interpretation of EWMA and p-chart methods over CUSUM techniques. Accordingly, CUSUM methodologies may be less appropriate for providing visual performance feedback to healthcare providers. Overall, the EWMA was found to be best suited for detecting outliers in non-indicated bone scan rates and disseminating that information to urology practices as a means of feedback. However, the EWMA is not a one-size-fits-all solution; for different applications and conditions one of the other control chart methodologies may very well be more appropriate or have higher resolution.

Very few studies have investigated the use of SPC methods in urology. Sood et al. employed Bernoulli CUSUM to continuous learning curve analysis in robotic kidney transplantation with regional hypothermia, using Shewhart control charts to monitor technical and functional outcomes such as anastomosis time and renal function [6]. By using SPC chart monitoring, they were able to demonstrate that functional outcomes were preserved despite longer anastomosis times in surgeons with less experience. In monitoring morbidity and mortality in radical cystectomy using Bernoulli CUSUM, Chalasani et al. found CUSUM’s ease of use and interpretation for continual outcome monitoring to be acceptable [14]. It is worth noting that our assertion that EWMA is easier to use and interpret than CUSUM does not conflict with this finding. Given the differing priorities in continual monitoring for patient safety, it is reasonable that different SPC methods were found to be optimal. In particular, individual patient case-by-case monitoring is well suited to Bernoulli CUSUM. Such a method may be helpful for monitoring sepsis following prostate biopsy.

The four control chart methodologies’ characteristics are summarized in Table 2. Graphical interpretation reflects the degree to which chart values correspond to actual imaging rates; the CUSUM statistic is rather abstract and thus harder to interpret. Past studies have shown a relatively modest difference in performance between EWMA and CUSUM, and both types of charts are known to outperform the p-chart [15, 16]. While both the p-chart and CUSUM methodologies are more commonly used in hospital and healthcare surveillance settings than EWMA, it is appropriate to consider the EWMA’s nonstatistical benefits of ease of setup and interpretation when selecting a control chart methodology [17, 18].

**Table 2:** Comparison of control chart methodologies

	Graphical Interpretation	Average Run Length Performance
P-chart	*****	*
Weighted Binomial CUSUM	**	****
Bernoulli CUSUM	**	****
EWMA	****	*****

Comparison of the control chart methodologies across the domains of ease of interpretation and average run length performance, with five stars being best and one star being worst.

This study has several limitations. First, while SPC techniques are commonly calibrated using hundreds, if not thousands, of time periods worth of data points, the 6 years of MUSIC data available made it difficult to set up control charts using parameters matched to actual patient data. Given that the MUSIC imaging rates were not in steady state throughout the study period due to the intervention to reduce non-indicated imaging, Monte Carlo simulation was the best option to evaluate the different control chart methodologies with a great degree of statistical significance. Second, the wide variation in MUSIC practice sizes is not captured by the Monte Carlo simulation data modeled after a single representative practice. However, each control chart methodology employs measures that account for sample size and its variation in a statistically robust way. Lastly, the EWMA’s ARL of 5.5 quarters (1.4 years) appears at first glance to be an unreasonable length of time to allow before detecting change. This time period, however, is a reflection of the control chart’s ability to distinguish statistical noise from poor performance and avoid false positive signals. Of note, the EWMA identifies increased non-indicated imaging more than three times faster than the p-chart.

These limitations notwithstanding, our findings show that the EWMA control chart alerts its user to process changes in a timely manner while maintaining a low rate of Type 1 error, all while being relatively easy to interpret by physicians. Our work also shows that the p-chart is easy to interpret but takes a significantly greater length of time to detect change. Our findings may inform implementation of automated systems for monitoring various forms of attribute data (such as guideline adherence or complications) and providing rapid feedback. Such systems may be useful in identifying outlying performance among hospitals, urology practices, or individual physicians and in doing so help capitalize on opportunities for improvement. To this end, MUSIC is in the process of implementing the EWMA method to monitor imaging guideline adherence.

## Conclusions

We showed how Monte Carlo simulation can be used to compare performance of control chart methodologies in the context of a quality improvement collaborative. Overall, the EWMA chart provides feedback significantly earlier than the Bernoulli CUSUM, p-chart, and weighted binomial CUSUM methods. The p-chart and EWMA are much easier to interpret than CUSUM charts. Due to its superior performance, high ease of setup, and clarity of interpretation, we determined the EWMA to be the most suitable SPC technique for monitoring imaging guideline adherence.

## Supplementary information

**Additional file 1.** Supplemental Methods. Contains further detailed discussion of relevant statistical process control methods and Monte Carlo simulation

**Additional file 2.** Ethics Statement Supplement. Contains details of MUSIC practices’ IRB status.

## Data Availability

The datasets generated and analysed that support the findings of this study are available on request from the corresponding author [K.G.] on reasonable request. The data are not publicly available due to institutional policy of data handling (containing information that could compromise research participant privacy/consent).

## Abbreviations

ARL: Average run length
CUSUM: Cumulative sum
EWMA: Exponentially weighted moving average
MUSIC: Michigan Urological Surgery Improvement Collaborative
PSA: Prostate specific antigen
SPC: Statistical process control
