# Supplementary material for: Prospective monitoring of imaging guideline adherence by physicians in a surgical collaborative: comparison of statistical process control methods for detecting outlying performance
Source: BMC Med Inform Decis Mak. 2020 May 13;20:89. doi: 10.1186/s12911-020-1126-z (PMC7218839; doi:10.1186/s12911-020-1126-z)
Supplement: Supplementary file 1 — Additional file 1. Supplemental Methods. Contains further detailed discussion of relevant statistical process control methods and Monte Carlo simulation [file 12911_2020_1126_MOESM1_ESM.docx]

**Supplemental Methods**In a control chart, performance measurements are charted relative to a center line representing where the measurement should fall, an upper control limit (UCL), and a lower control limit (LCL). Should a measurement fall outside the range of the control limits, the process is deemed out-of-control and an alarm is signaled, indicating a need for corrective action. An intervention takes place to restore the process to its usual, in-control state and the chart is reset.[1, 2]

*P-Chart*
The p-chart is one of the simplest control charts used for attribute data. The proportion $p_{i}$ of patients with non-indicated imaging in the $i$^th^ quarter is plotted and compared with a center line $\bar{p}$ representing a target value. An alarm is triggered when the charted proportion $p_{i}$ falls above the UCL, indicating excessive non-indicated imaging, or below the LCL, indicating a decrease in the rate of non-indicated imaging. The UCL and LCL are typically defined as being $L$ multiples of the sample standard deviation $\sigma_{i}$ from the center line, as shown in equations 1-4 below. The sample standard deviation varies between quarterly samples in a manner dependent on $n_{i}$, the number of patients non-indicated for imaging in the $i$^th^ quarter. While $L$ is customarily given a value of 3, the width of control limits may be changed as a matter of judgement if, for example, greater sensitivity to shifts in process mean is desired.[3]

$$\begin{aligned} {UCL}_{i}= \bar{p}+L\sigma_{i}\#\left( 1 \right) \end{aligned}$$

$$\begin{aligned} Center line= \bar{p}\#\left( 2 \right) \end{aligned}$$

$$\begin{aligned} {LCL}_{i}= \bar{p}+L\sigma_{i}\#\left( 3 \right) \end{aligned}$$

$$\begin{aligned} \sigma_{i}=\sqrt{\frac{\bar{p}\left( 1-\bar{p} \right)}{n_{i}}}\#\left( 4 \right) \end{aligned}$$

The control limits described above are valid only as long as the criteria of both equations 5 and 6 hold true; otherwise the control limits must be calculated using exact limits found via the probability distribution function of the binomial distribution.[3]

$$\begin{aligned} n_{i}\bar{p}\left( 1-\bar{p} \right)>5\#\left( 5 \right) \end{aligned}$$

$$\begin{aligned} 0.1< \bar{p}< 0.9\#\left( 6 \right) \end{aligned}$$

*Exponentially weighted moving average (EWMA) Chart*
Unlike the p-chart, which evaluates data from one quarter at a time in determining whether the process mean has deviated substantially from the target value, the EWMA chart takes into account all data to date using the EWMA statistic $z_{i}$ defined in Equation 7, with greater weighting for more recent samples.[4]

$$\begin{aligned} z_{i}=\lambda p_{i}+\left( 1-\lambda\right)z_{i-1}\#\left( 7 \right) \end{aligned}$$

$\lambda$ is a constant between 0 and 1 which determines the weighting of the EWMA statistic. A larger value of $\lambda$ results in the EWMA statistic weighting recent values $p_{i}$ more highly. The starting value $z_{0}$ is set to be equal to $\bar{p}$, the chart’s target value which is also the value of the center line. An alarm is triggered when the EWMA statistic $z_{i}$ falls above or below the UCL or LCL, indicating deterioration or improvement in performance respectively. Due to the EWMA’s dependence on past data, its control limits depend not only on the number $n_{i}$ of patients non-indicated for imaging in the current quarter but also those of past quarters, as shown in equations 8-10 below.

$$\begin{aligned} {UCL}_{i}= \bar{p}+L\lambda\sqrt{\bar{p}\left( 1-\bar{p} \right)}\sum_{j=1}^{i} \frac{\left( 1-\lambda\right)^{2\left( i-j \right)}}{n_{j}}\#\left( 8 \right) \end{aligned}$$

$$\begin{aligned} Center line= \bar{p}\#\left( 9 \right) \end{aligned}$$

$$\begin{aligned} {LCL}_{i}= \bar{p}-L\lambda\sqrt{\bar{p}\left( 1-\bar{p} \right)}\sum_{j=1}^{i} \frac{\left( 1-\lambda\right)^{2\left( i-j \right)}}{n_{j}}\#\left( 10 \right) \end{aligned}$$

Choices of L and $\lambda$ are a design choice dependent on the desired average run length properties and the expected size of the shift in process mean, and are discussed in detail by Lucas and Saccucci.[5] In this instance, 0.2 was used as the value for $\lambda$. The EWMA is insensitive to the assumption that the data are normally distributed and is known to be a good alternative to the p-chart for detecting small shifts in a process mean.[4] In addition, the EWMA statistic can be regarded as a forecast for the next data point in the process.[6]

*Cumulative sum (CUSUM) Overall Summary*
CUSUM is another control chart method that takes into account both present and past samples’ data. It is known to be more sensitive to small shifts in process mean than Shewhart control charts, but is not as simple to set up or to interpret.[7] In addition, CUSUM does not clearly indicate the level of current performance beyond whether or not the process is nearing a control limit. CUSUM is well suited for phase II applications of statistical process control where large amounts of process data have already been gathered, as it requires the standard deviation of the process to be known or to have a reliable estimate.[4]

In the CUSUM control chart method, the observed value from each sample has a target value subtracted from it. The resulting difference is summed cumulatively and plotted; Equation 11 illustrates the calculation of the CUSUM statistic $C_{i}$ for the case of a fixed sample size.[4]

$$\begin{aligned} C_{i}= \sum_{j=1}^{i} (p_{i}-\bar{p})\#\left( 11 \right) \end{aligned}$$

When the process mean is in-control, deviations above the target value and below the target value will tend to cancel each other out, resulting in the chart progressing in a relatively flat manner. However, if the process deviates off target, these differences will accumulate and the chart will develop an upward or downward slope.

In order to determine when an alarm should be signaled to indicate that the process mean has deviated from the target, we use the tabular CUSUM. Deviations above the target are evaluated with the upper CUSUM statistic and deviations below the target are evaluated with the lower CUSUM statistic as shown in equations 12 and 13 respectively[8], wherein $C_{0}^{+}=C_{0}^{-}=0$.

$$\begin{aligned} C_{i}^{+}=\max\left[ 0, {C_{i-1}^{+}+p}_{i}-\bar{p}-k \right]\#\left( 12 \right) \end{aligned}$$

$$\begin{aligned} C_{i}^{-}=\min\left[ 0, {C_{i-1}^{-}+p}_{i}-\bar{p}+k \right]\#\left( 13 \right) \end{aligned}$$

The reference value $k$ is determined by the size of the anticipated shift in the process mean and acts to draw the CUSUM statistics toward zero. In the upper CUSUM an alarm is signaled if the upper CUSUM statistic $C_{i}^{+}$ exceeds upper control limit $h^{+}$, while in the lower CUSUM an alarm is signaled if the lower CUSUM statistic $C_{i}^{-}$ falls below the lower control limit $h^{-}$. The control limits $h$ are determined based on the desired sensitivity of the CUSUM chart. Smaller values of $h$ result in greater sensitivity, but with a corresponding increase in the rate of false alarms.

*Weighted Binomial CUSUM Chart*The imaging appropriateness data consists of patients aggregated quarterly. Under the assumption that each patient is selected independently from the same probability distribution, where they each have a chance of receiving non-indicated imaging or not receiving non-indicated imaging, the binomial distribution is an appropriate model for quarterly imaging appropriateness data.[9] The variability in the number of prostate cancer patients in any given quarter necessitates that the data from different quarters be weighted accordingly. While CUSUM is typically oriented toward the analysis of normally distributed data of fixed sample size, Hawkins and Olwell teach the weighted binomial CUSUM method, which is a minor adjustment of CUSUM to properly monitor binomial data with variable sample size.[9] A modification to the tabular CUSUM statistics is made, as shown in equations 14 and 15 wherein $k_{u}$ is a per unit reference value defined in equations 16 and 17 and is dependent on the in-control probability $\bar{p}$ and the upper or lower out of control probability $p^{+}$ or $p^{-}$ respectively.

$$\begin{aligned} C_{i}^{+}=\max\left[ 0, {C_{i-1}^{+}+p}_{i}-n_{i}k_{u}^{+} \right]\#\left( 14 \right) \end{aligned}$$

$$\begin{aligned} C_{i}^{-}=\min\left[ 0, {C_{i-1}^{-}+p}_{i}-n_{i}k_{u}^{-} \right]\#\left( 15 \right) \end{aligned}$$

$$\begin{aligned} k_{u}^{+}=-\frac{\ln\left( \frac{1-p^{+}}{1-\bar{p}} \right)}{\ln\left( \frac{p^{+}\left( 1-\bar{p} \right)}{\bar{p} \left( 1-p^{+} \right)} \right)}\#\left( 16 \right) \end{aligned}$$

$$\begin{aligned} k_{u}^{-}=-\frac{\ln\left( \frac{1-p^{-}}{1-\bar{p}} \right)}{\ln\left( \frac{p^{-}\left( 1-\bar{p} \right)}{\bar{p} \left( 1-p^{-} \right)} \right)}\#\left( 17 \right) \end{aligned}$$

*Bernoulli CUSUM Chart*The Bernoulli CUSUM method uses individual counts of attribute data as input. In evaluating non-indicated imaging rates, individual patients are counted as ‘1’ if they received inappropriate imaging and ‘0’ if they correctly did not receive imaging. By evaluating patients on an individual basis, Bernoulli CUSUM has the advantage of being able to signal alarm without having to wait for completion of an entire sample; additionally, the Bernoulli CUSUM method is capable of detecting large shifts in process mean faster than binomial CUSUM methods.[10] The Bernoulli CUSUM is a special case of the weighted binomial CUSUM wherein rather than representing quarters, each sample represents a single patient so $n_{i}=1$ for all $i$.

$$\begin{aligned} C_{i}^{+}=\max\left[ 0, {C_{i-1}^{+}+p}_{i}-k_{u}^{+} \right]\#\left( 18 \right) \end{aligned}$$

$$\begin{aligned} C_{i}^{-}=\min\left[ 0, {C_{i-1}^{-}+p}_{i}-k_{u}^{-} \right]\#\left( 19 \right) \end{aligned}$$

*Standardization of control charts for Monte Carlo simulation*When a process is in the in-control state, a high average run length (ARL) is desirable, as it is indicative of a low rate of false alarm, or Type 1 error. In the out-of-control state, a low ARL is desirable because it indicates that the control chart signals an alarm rapidly when the process mean shifts, whereas a high ARL would indicate that the control chart is relatively insensitive to the out-of-control state.

Comparison of the control charts using the Monte Carlo method was performed by using simulated data at an in-control level to set chart parameters to standardize the different charts to have an approximate 10% false signal rate over five years, i.e. a given practice with in-control performance would have a 10% overall chance of having a control chart signal falsely. This value was subjectively selected as a rate of error low enough to prevent false-positive signals from being a nuisance. The control chart methodologies were then applied to simulated out-of-control data and the average run lengths were compared.

Parameters for Monte Carlo simulation of Michigan Urological Surgery Improvement Collaborative (MUSIC) practice data were based off the overall rates of non-indicated imaging within MUSIC and the number of patients seen by a representative MUSIC practice. The MUSIC overall non-indicated bone scan rate was 5.9% following the intervention, an improvement from 11.4% preceding the intervention. Accordingly, 5.9% was used as the in-control non-indicated imaging rate and 11.4% was used as the out-of-control rate of the Monte Carlo simulation in order to approximate actual MUSIC overall performance. Among practices with over 40 new diagnoses of prostate cancer per year, the mean MUSIC practice by patient volume had an average of 26 patients per quarter (3-month period), and this value was accordingly used as the control chart sample size for each quarter.

A set of in-control data was generated as a sequence of binomial random variables with size 26 and probability 5.9%. Each control chart methodology was applied to this set of data to tune the chart parameters. Chart parameters were adjusted manually to standardize the different control charts to a 10% five-year false signal rate.

For the p-chart and EWMA, the value $L$ determining the width of the control limits was adjusted manually until the five-year false signal rate was approximately 10%. Different values of L were used for the two different control chart methodologies.

CUSUM chart parameters were determined by setting the out-of-control limit $p^{+}$ so as to be 1 standard deviation above the center line and for $p^{-}$ to be set below the centerline with the same proportion as the center line relative to $p^{+}$, as described in equations 20 and 21 wherein center line $\bar{p}=.059$ and number of patients per quarter $n=24$ for every quarter.

$$\begin{aligned} p^{+}=\bar{p}+\sqrt{\frac{\bar{p}\left( 1-\bar{p} \right)}{n}}\#\left( 20 \right) \end{aligned}$$

$$\begin{aligned} p^{-}=\bar{p}\frac{\bar{p}}{p^{+}}\#\left( 21 \right) \end{aligned}$$

Reference values $k_{u}$ were calculated as described in equations 18 and 19. Decision limits $h^{+}$ and $h^{-}$ were adjusted manually in order to obtain the desired one-year false signal rate.

Following determination of control chart parameters, average run length for each control chart methodology using those parameters was determined at the 6% through 11% levels. Average run lengths were determined by applying each control chart to the simulated data so as to generate a signal 10,000 times and taking the arithmetic mean of the number of quarters that had elapsed prior to signaling. ARLs were compared using Tukey’s Honestly Significant Differences test.

**References**

**1.** Page ES. Cumulative Sum Charts. *Technometrics.* 1961;3:1-&.

**2.** Gan FF. Designs of one- and two-sided exponential EWMA charts. *J Qual Technol.* 1998;30:55-69.

**3.** Mohammed MA, Worthington P, Woodall WH. Plotting basic control charts: tutorial notes for healthcare practitioners. *Quality & safety in health care.* 2008;17:137-145.

**4.** Montgomery DC. *Introduction to Statistical Quality Control*. Hoboken, NJ: Wiley; 2008.

**5.** Lucas JM, Saccucci MS. Exponentially Weighted Moving Average Control Schemes - Properties and Enhancements. *Technometrics.* 1990;32:1-12.

**6.** Lucas JM, Crosier RB. Fast Initial Response for Cusum Quality-Control Schemes - Give Your Cusum a Head-Start. *Technometrics.* 1982;24:199-205.

**7.** NIST/SEMATECH e-Handbook of Statistical Methods.

**8.** Hawkins DM, Olwell DH. *Cumulative sum charts and charting for quality improvement*. New York: Springer; 1998.

**9.** Hawkins DM, Olwell DH. Cumulative sum charts and charting for quality improvement. *Statistics for engineering and physical science*. New York: Springer; 1998:105-134.

**10.** Reynolds MR, Stoumbos ZG. A general approach to modeling CUSUM charts for a proportion. *Iie Trans.* 2000;32:515-535.

**Abbreviation list**

ARL: average run length; CUSUM: cumulative sum; EWMA: exponentially weighted moving average; LCL: lower control limit; MUSIC: Michigan Urological Surgery Improvement Collaborative; UCL: upper control limit
